# The Effect of Szigetvár Medicinal Water on HaCaT Cells Exposed to Dithranol

**DOI:** 10.3390/life14101318

**Published:** 2024-10-17

**Authors:** István Szabó, Ágnes Szenczi, Afshin Zand, Tímea Varjas, Csaba Varga

**Affiliations:** Department of Public Health Medicine, Medical School, University of Pécs, 7622 Pécs, Hungary; szenczi.agnes@gmail.com (Á.S.); afshin.zand@aok.pte.hu (A.Z.); timea.varjas@aok.pte.hu (T.V.); drvargacsababt@gmail.com (C.V.)

**Keywords:** medicinal water, thermal water, dithranol, organic substances, psoriasis, oxidative stress, MDA, pro-inflammatory cytokine

## Abstract

(1) Introduction: Topical dithranol is still commonly used today as an effective treatment for psoriasis. Dithranol treatment is often supplemented with balneotherapy, which has been shown to increase effectiveness and reduce side effects. The inorganic salts (sulfhide, selenium, zinc) are usually thought to be responsible for the effect. The antioxidant effect of the waters is thought to be behind the therapeutic effect, for which inorganic substances (sulfides, selenium, zinc) are thought to be responsible. The organic matter content of medicinal waters is also particularly important, as humic acids, which are often found in medicinal waters, have antioxidant effects. (2) Methods: In this short-term experiment, we aimed to test the possible protective effect of Szigetvár medicinal water and its organic matter isolate on HaCaT cells exposed to dithranol. Malondialdehyde levels were measured, and RT-qPCR was used to investigate the gene expression of selected cytokines relevant in the oxidative stress response (IL-6, IL-8, TNF-α, GM-CSF) and the expression of microRNA-21. (3) Results: Szigetvár medicinal water and the organic isolate prevented the increase in malondialdehyde levels caused by dithranol treatment. The cytokine gene expressions elevated by dithranol exposure were reduced by the treatment. (4) Conclusions: Szigetvár medicinal water and organic substances alone may have a protective effect on patients’ healthy skin surfaces against dithranol damage. We also demonstrated that the organic compounds are also responsible for the protective effect.

## 1. Introduction 

Psoriasis, a common chronic immune-mediated condition, affects approximately 1–3% of the general population [1]. Despite its well-known side effect profile, topical dithranol (DTH) is still used in many places for the treatment of psoriasis. Dithranol (DTH) is an effective agent for treating psoriasis; however, it also causes oxidative damage to healthy skin areas, so the risk–benefit should be carefully considered [2,3,4]. DTH undergoes chemical reactions that produce reactive oxygen species (ROS), causing inflammation of the psoriatic plaque; this is considered a necessary mode of action. In clinical practice, DTH treatment is applied in several short-contact treatments [5]. The initial effects of DTH can be observed by examining the expression of various pro-inflammatory cytokines, including interleukin-6 (IL-6), IL-1, IL-8, and tumor necrosis factor-alpha (TNF-α). Additionally, the expression of granulocyte-macrophage colony-stimulating factor (GM-CSF) can indicate the infiltration of immune cells [6,7]. Different treatment methods often support topical DTH treatment; for example, spa hospitals and spa services offer balneotherapy to promote healing and counteract the inflammatory side effects on intact skin areas [8,9,10]. Balneotherapy involves healing practices conducted by bathing, drinking, or inhaling natural medicinal waters, peloids, or gases [11,12,13]. Practice and several randomized controlled trials proved the effectiveness of balneotherapy. Mechanical, thermal, and chemical effects, as well as their combinations, are responsible for the healing effect [14,15]. Balneotherapy in treating psoriasis has been documented for a century and a half and is still part of medical practice today [16,17]. Dissolved minerals, their adsorption through intact skin, and their involvement in the therapeutic response are some of the critical working mechanisms behind the effect of balneotherapy [14,18,19]. Many medicinal waters contain a remarkable amount of organic matter. The absorption of organic components might correlate with certain beneficial effects of balneotherapy [20,21,22,23,24,25]. Humic and fulvic acids are common organic compounds in thermal waters. Interestingly, many compounds from the HA fraction have remarkable antioxidant and radioprotective effects [26,27]. This research is based on the previous experiments of our working group in which medicinal waters, peloids, and their organic isolates have been tested on prokaryotic systems, cell cultures, and human trials [28,29,30,31]. On UV-exposed HaCaT cells, we used the comet assay test to evaluate numerous thermal waters and their organic matter isolates. We proved that many of them, including the organic isolates, decreased DNA fragmentation [28]. In a pilot study, Szenczi et al. found that complementary balneotherapy treatment for psoriasis could protect intact skin areas of patients from undesirable oxidative stress caused by DTH therapy [10]. The potential healing effects of Szigetvár thermal water and organic matter isolate were previously highlighted and proved in a clinical trial [29]. In the present experiment, we aimed to investigate the effect of Szigetvár medicinal water on HaCaT cells exposed to DTH in a short-term protocol (1 h of DTH exposure followed by 3 h of balneotherapy). We tested Szigetvár medicinal water, which contains all organic and inorganic substances. We also tested the organic isolate of Szigetvár medicinal water, which is free of salt content. We assumed that Szigetvár medicinal water may have a protective effect against oxidative stress caused by DTH, which may protect intact skin areas exposed to the inflammatory side effects of DTH. We also assumed that the organic isolate may act similarly to the whole water, thus demonstrating that the therapeutic effect is not solely due to inorganic substances. Human keratinocyte cell line (HaCaT) was exposed to different concentrations of DTH, cell culture medium made from Szigetvár medicinal water (SVNM), and medium supplemented with organic isolate to determine the possible protective effect against DTH-induced oxidative stress. Malondialdehyde (MDA) concentrations were measured as a marker of oxidative stress [10,32,33,34]. Using the same protocol, we also determined the mRNA expression of key cytokines (IL-6, IL-8, TNF-α, GM-CSF) in the early response to oxidative stress to analyze the effect of DTH exposure [35,36,37]. We also measured the expression of microRNA-21 (miRNA, miR-21), which plays a crucial role in oxidative stress and apoptosis [38,39,40].

## 2. Materials and Methods

### 2.1. Cell Culture and Experimental Media

HaCaT cells [41] (CLS Cell Lines Service GmbH, Eppelheim, Germany) were routinely cultured according to the manufacturer’s recommendations in Dulbecco’s modified Eagle’s medium (DMEM, Merck-Sigma-Aldrich, Budapest, Hungary) supplemented with 10% Fetal Bovine Serum (FBS, Capricorn Scientific, Ebsdorfergrund, Germany) and 1% penicillin/streptomycin solution (Merck-Sigma-Aldrich, Budapest, Hungary), incubated in 100% relative humidity and 5% CO_2_ atmosphere. We exposed cells to normal cell culture medium supplemented with Szigetvár water organic matter isolate (isolate), and cell culture medium prepared from Szigetvár thermal water (SVNM). The isolate was prepared as described by Hanzel et al. [29]. The final organic matter concentration of the isolate was 3000 times that of the original water based on the elution rate. The isolate contained 50% ethanol. To select the optimal isolate concentration for the experiments, the isolate was diluted with DMEM to achieve dilutions of 6×, 12×, 30×, 40×, 60×, 120×, 250×, 300×, 400×, 600×, 1500×, and 3000× of the isolate. The composition of the dilution series tested is shown in (Table 1). To select the ideal amount of Szigetvár water in the SVNM, a dilution series of Szigetvár water and ultrapure water was prepared in 10% steps between 10 and 90%. DMEM powder (Merck-Sigma-Aldrich, Budapest, Hungary), used at 12.4 g/L with the addition of 3.7 g/L NaHCO_3_ (Molar Chemicals, Budapest, Hungary), with 10% FBS and 1% penicillin/streptomycin solution, was added to obtain a complete cell culture medium. We tested all isolates and SVNM dilutions in the XTT cell viability assay [42]. Based on the results of the cell survival tests, we chose SVNM with 90% Szigetvár water content and isolates at 120×, 600×, and 3000× dilutions for our RT-qPCR and MDA experiments. The 3000× dilution of the isolate is identical to the SVNM in terms of organic matter content, but without the original salt content. To prepare DTH containing cell culture medium, DTH (Molar Chemicals, Budapest, Hungary,) was dissolved in dimethyl-sulfoxide (DMSO, Molar Chemicals, Budapest, Hungary). This solution was added to DMEM (0.5% FBS content) to obtain final concentrations of 0.1, 0.25, and 0.5 μg/mL. High-power ultrasonic agitation and stirring were used to promote dissolution. All DTH experimental media were freshly prepared prior to the experiment.

### 2.2. XTT Cell Viability Assay

The HaCaT cells were tested for viability using the Invitrogen Cyquant XTT Cell Viability Assay Protocol (Thermo Fischer Scientific, Budapest, Hungary). The experiments were conducted in 96-well cell culture plates (VWR, Budapest, Hungary). The plates were seeded with 1 × 10^4^ cells/cm^2^, and the experiment was conducted when 50–60% confluence was reached. Seeding cell density was measured with a Bio-Rad TC20 automated cell counter (Bio-Rad Laboratories, Hercules, CA, USA). The XTT assays were read with a Dialab Diareader ELX-800g-PC plate reader (Dialab, Budapest, Hungary) according to the manufacturer’s protocol. All the SVNM and isolate treatments lasted for 3 h as described in the experimental protocol for the MDA and RT-qPCR experiments (Table 2). The survival percentage was calculated based on the results of untreated (control) cells, which were assumed to be 100% survival. We replicated every experimental setting three times.

### 2.3. Experiment Protocol for RT-qPCR and MDA Assay

Based on the survival test results, we used SVNM containing 90% Szigetvár water and 120×, 600×, and 3000× dilutions of the organic isolate (abbreviated as 120×, 600×, and 3000×). The experiment protocol is represented in Table 2. DTH treatment was applied for 1 h in all cases. For DTH 1-h exposure (Treatments #8, #14, and #20, DTH-1 h), we washed and harvested the cells right after 1 h. For the DTH 4-h exposure exposure (Treatments #9, #15, and #21, DTH-4 h), 1 h of DTH exposure followed by 3 h of DMEM treatment was applied. All the SVNM and isolate treatments lasted for 3 h.

### 2.4. Lipid Peroxidation (MDA) Assay

The quantification of MDA levels was performed using a Lipid Peroxidation (MDA) Assay Kit (Merck-Sigma-Aldrich, Budapest, Hungary). The kit relies on a colorimetric reaction involving MDA and thiobarbituric acid. The reaction product’s absorbance is proportional to the MDA concentration. The reactions were carried out according to the manufacturer’s protocol. In each sample, 2 × 10^6^ cells were measured. Spectrophotometric reading at 532 nm was carried out on a Berthold LB 915 Colibri Microvolume Spectrometer (Berthold Technologies GmbH, Bad Wildbad, Germany), using a blank described in the protocol. Concentrations were determined based on a standard concentration curve parallel to the measurements. We replicated every experimental setting five times. All three concentrations of vehicle controls were prepared with the same amount of DMSO and 50% ethanol (Molar Chemicals, Budapest, Hungary) as the experimental media. Since we did not find significant differences between the DMSO (1 and 4 h exposure) or ethanol controls, we only represented the vehicle controls for the 600× dilution and the 0.25 μg/mL DTH in the figure.

### 2.5. Quantitative Real-Time Polymerase Chain Reaction (qRT-PCR)

**Figure 1:**
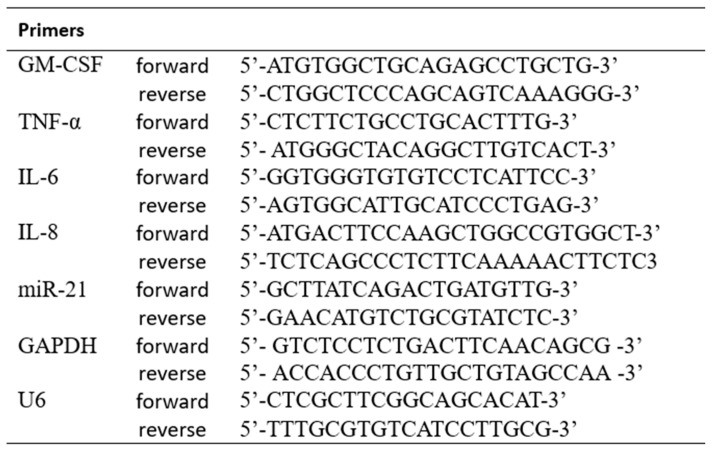
The sequence of the primers used in the experiment.

The experiment protocol was carried out in 96-well cell culture dishes. The cells were directly harvested, and the RNA was isolated using the ExtraZol reagent (Qiagen, Hilden, Germany) protocol. The total RNA concentration was measured with a Maestrogen MN-913 spectrophotometer (Maestrogen, Hsinchu, Taivan). Primers were designed using the NCBI Primer-BLAST (using Primer3web software version 4.1.0). The primers were purchased from Integrated DNA Technologies (IDT, Coralville, Iowa, USA). The sequence of the primers is shown in Figure 1. The PCR was performed on a LightCycler 480 PCR system (Roche, Budapest, Hungary) using the qPCRBIO SyGreen 1-step Detect Lo-ROX kit (Nucleotest Bio, Budapest, Hungary). The reaction setup was the following: 10 µL qPCRBIO SyGreen 1-Step Mix, 0.8 µL forward primer, 0.8 µL reverse primer, 1 µL RTase Go (RNAse inhibitor), samples with 1 pg–10 ng total RNA concentration, PCR grade water up to 20 µL final volume. After 10 min at 50 °C reverse transcription and 2 min at 95 °C polymerase activation, 40 two-step amplification cycles were carried out at 95 °C for 5 s and 65 °C for 20 s. The expression levels of miRNA-21 and IL-6, IL-8, TNF-α, and GM-CSF were analyzed by the 2^−∆∆Ct^ method and normalized to the internal controls U6 (for miRNA-21) and GAPDH (for mRNA). We replicated every experimental setting three times. All three concentrations of vehicle controls were prepared with the same amount of DMSO and 50% ethanol as the experimental media. Since we did not find significant differences between the DMSO (1 and 4 h exposure) or ethanol controls at any of the measured cytokines or in the case of miR-21, we only represented the vehicle controls for the 600× dilution and the 0.25 μg/mL DTH in the figures.

### 2.6. Statistical Analysis

We used the Kruskal–Wallis one-way ANOVA and the Student’s *t*-test to analyze our results statistically. The significance level was set to *p* < 0.05. Significant changes are marked with an asterisk (*) in the figures. The statistical analysis was performed with IBM SPSS Statistics Version 26.0 for Windows (Armonk, NY, USA).

## 3. Results

### 3.1. Cell Viability in SVNM and Organic Isolates

**Figure 2:**
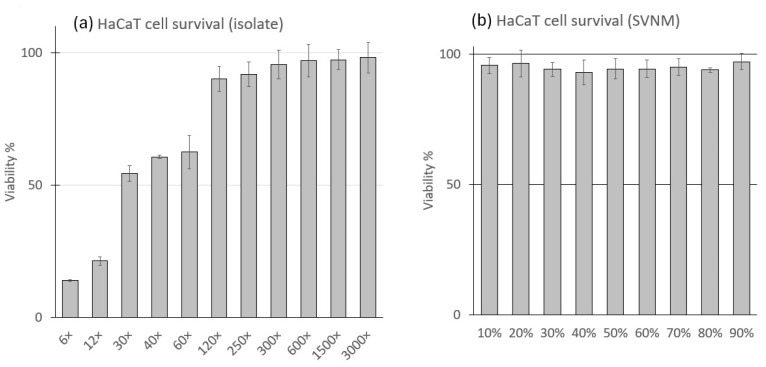
(**a**) Survival of HaCaT cells in different dilutions of Szigetvár organic matter isolate in 3 h. (**b**) Survival of HaCaT cell in different concentrations of Szigetvár medicinal water containing cell culture medium (SVNM). 6×–3000×–dilution of the isolate (Composition shown in Table 1) in 10–90% Szigetvár medicinal water content of different studied SVNMs.

A series of SVNM containing 10–90% Szigetvár water in 10% steps has been tested on the viability of HaCaT cells using the XTT cell viability assay for 3 h. No significant change in viability has been measured; cells showed neither morphological changes nor loss of viability at any concentration. This experimental series aims to investigate the short-term effects of DTH treatment over 4 h (1 h of DTH treatment and 3 h of balneotherapy The isolate was diluted with DMEM to achieve dilutions of 6×, 12×, 30×, 40×, 60×, 120×, 250×, 300×, 400×, 600×, 1500×, and 3000× of the isolate. These dilutions were applied to HaCaT cells for 3 h, and after exposure, viability was tested with the XTT cell viability assay for 4 h. The results of the cell viability test are shown in Figure 2. Our results show that 6×−60× dilutions significantly decreased the viability of the HaCaT cells during 3 h of treatment. The first line that reached 90% viability was 120× dilution. At lower isolate dilutions, the higher cell lethality was likely influenced by the excessively high concentration of the vehicle (ethanol). As we focused on the overall effect of the complete isolate, we did not examine the effect of the vehicle separately. Compared to the control, higher dilutions than 120× did not significantly change viability. The cells under the microscope were investigated, and morphological changes (granulation, detachment, loss of shape) were observed among those treated with the 6×−60× dilutions. No morphological changes were observed at 120× dilution and higher. 

### 3.2. Lipid Peroxidation (MDA) Assay

**Figure 3:**
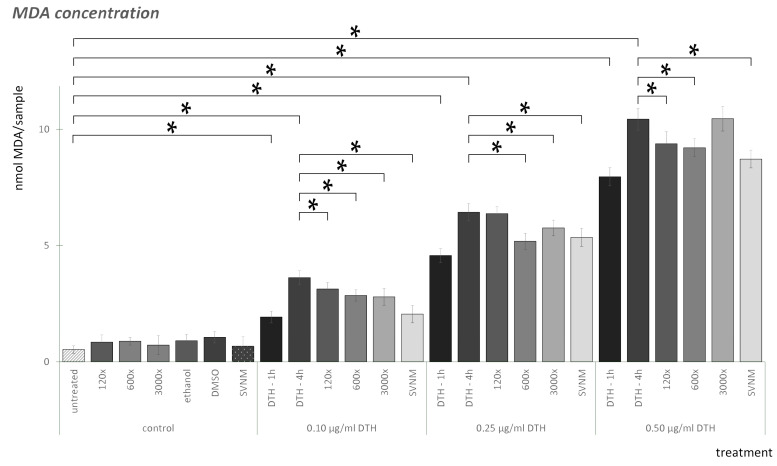
Amount of MDA (nmol/sample) in HaCaT cells in different experimental settings (See Table 2). (* = *p* < 0.05).

The results of the MDA assay are shown in Figure 3. Checking all the negative controls, neither SVNM, 120×, 600×, nor 3000× dilutions elevated MDA levels. The vehicle controls (ethanol, DMSO) did not elevate MDA levels significantly compared to the negative control. We did not find significant differences between the DMSO controls at the three concentrations examined. We did not find significant differences between the ethanol controls. DTH elevated MDA levels in a concentration-dependent manner. The increase is already significant after one hour for all three concentrations; however, after four h of DTH exposure, the increase is even more noticeable. The 120× dilution of the organic isolate significantly decreased MDA levels in the case of 0.1 and 0.5 µg/mL DTH concentrations compared to the DTH-4 h exposure. The 600× dilution significantly decreased MDA levels at all concentrations of DTH compared to the DTH-4 h exposure. The 3000× dilution also significantly decreased MDA levels at 0.1 and 0.25 µg/mL DTH concentrations compared to the DTH-4 h exposures. SVNM significantly reduced MDA levels at all DTH concentrations compared to the DTH-4 h exposures. If we compare the effect of SVNM and the dilutions, at 0.1 µg/mL SVNM is significantly lower than the dilutions, at 0.25 µg/mL SVNM is significantly lower than the 120× dilution, and at 0.5 µg/mL SVNM is significantly lower than the 3000× dilution. 

### 3.3. Relative Gene Expression of Selected Cytokines

**Figure 4:**
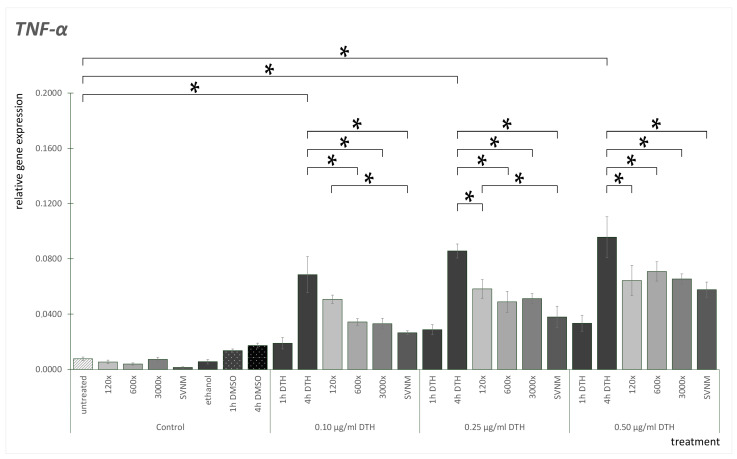
Relative gene expression level of TNF-α in HaCaT cells treated according to the experimental protocol. (* = *p* < 0.05). DTH—dithranol; 120×, 600×, 1200×-dilution of the tested organic matter isolate (Composition shown in Table 1); SVNM—Cell culture medium with 90% Szigetvár medicinal water content.

TNF-α mRNA expression results are shown in Figure 4. TNF-α mRNA expression was low in untreated cells. No significant change was observable when exposed to organic isolates. We did not find significant differences between the DMSO controls at the three concentrations examined. We did not find significant differences between the ethanol controls. When exposed to SVNM, the expression of TNF-α decreased. The DMSO control showed slightly elevated TNF-α mRNA expression. Compared to the DMSO control, 0.1 and 0.25 µg/mL DTH did not elevate, but 0.50 µg/mL DTH significantly elevated TNF-α expression in an h. Measuring 4 h positive DTH controls, all TNF-α mRNA expressions were significantly elevated compared to the DTH-1 h exposures. If we analyze the 0.1 µg/mL DTH treatment, we did not find any significant difference between the 4 h positive DTH control and the 120× dilution. However, 600× and 3000× dilution significantly decreased TNF-α mRNA expression. SVNM treatment also significantly decreased TNF-α mRNA expression. We found similar results in the case of the 0.25 µg/mL DTH samples: 120×, 600×, 3000×, and SVNM significantly decreased TNF-α mRNA expression compared to the 4 h positive DTH control. Analyzing the 0.5 µg/mL DTH samples, 120×, 600×, 3000×, and SVNM significantly decreased TNF-α mRNA expression compared to the DTH-4 h exposure. If we compare SVNM and dilutions, we only find a significant difference between 120× dilution and SNVM at 0.1 and 0.25 µg/mL DTH concentrations. 

**Figure 5:**
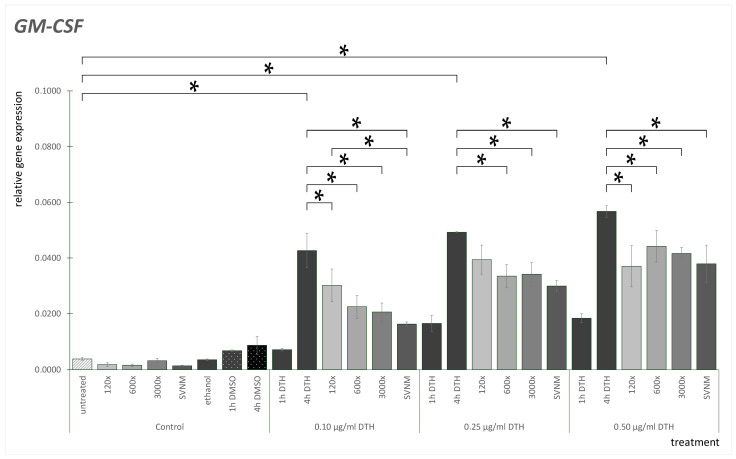
Relative gene expression level of GM-CSF in HaCaT cells treated according to the experimental protocol. (* = *p* < 0.05). DTH—dithranol; 120×, 600×, 1200×-dilution of the tested organic matter isolate (Composition shown in Table 1); SVNM—Cell culture medium with 90% Szigetvár medicinal water content.

The GM-CSF mRNA expression results are shown in Figure 5. GM-CSF mRNA expression was low in untreated HaCaT cells. Cells treated with 120×, 600×, or 3000× dilutions as well as SVNM did not elevate GM-CSF expression. We did not find significant differences between the DMSO controls at the three concentrations examined. We did not find significant differences between the ethanol controls. DTH treatment elevated GM-CSF mRNA expression significantly in the DTH-4 h exposure groups compared to the DTH-1 h exposure groups. 120×, 600×, and 3000× dilutions and SVNM treatment significantly decreased GM-CSF mRNA expression in all DTH concentrations, except that at 0.25 µg/mL DTH, 120× dilution caused no significant decrease compared to the 4 h DTH control. If we compare GM-CSF mRNA expression of 120×, 600×, 3000×, and SVNM to one another, we only find a significant difference in the 0.10 µg/mL group, which was treated with 120× dilution; the GM-CSF gene expression was significantly lower in the 0.1 µg/mL SVNM group.

**Figure 6:**
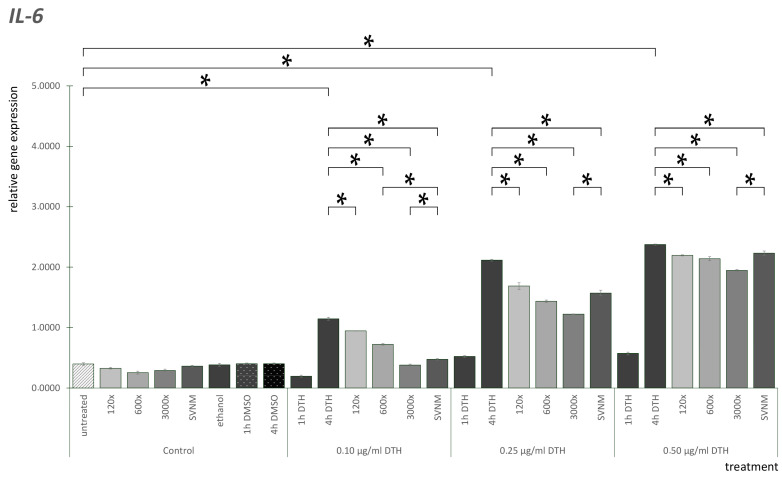
Relative gene expression level of IL-6 in HaCaT cells treated according to the experimental protocol. (* = *p* < 0.05). DTH—dithranol; 120×, 600×, 1200×—dilution of the tested organic matter isolate (Composition shown in Table 1.); SVNM—Cell culture medium with 90% Szigetvár medicinal water content.

The IL-6 mRNA expression results are shown in Figure 6. IL-6 mRNA expression was low in all the control groups. We did not find significant differences between the DMSO controls at the three concentrations examined. We did not find significant differences between the ethanol controls. After 4 h of DTH treatment, IL-6 expressions were significantly elevated dose-dependently. All dilutions and SVNM treatment decreased IL-6 overexpression significantly in all concentrations. If we analyze the difference between the balneotherapy treatments, in the case of the 0.1 µg/mL DTH groups, SVNM decreased IL-6 mRNA expression significantly compared to the 120× and 600× dilutions. The 3000× dilution was significantly lower than the SVNM. In the 0.25 µg/mL DTH groups, 3000× was significantly lower than the others. In the case of the 0.5 µg/mL DTH groups, 3000× dilution lowered IL-6 expression significantly compared to SVNM. 

**Figure 7:**
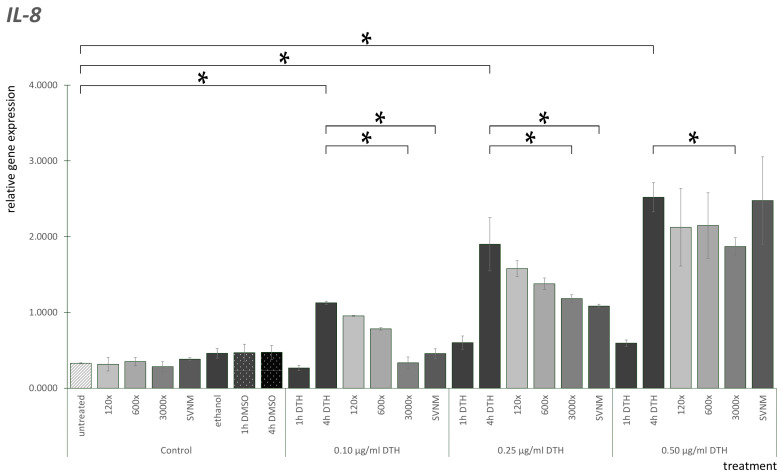
Relative gene expression level of IL-8 in HaCaT cells treated according to the experimental protocol. (* = *p* < 0.05). DTH—dithranol; 120×, 600×, 1200×-dilution of the tested organic matter isolate (Composition shown in Table 1); SVNM—Cell culture medium with 90% Szigetvár medicinal water content.

The IL-8 mRNA expression results are shown in Figure 7. IL-8 mRNA expression was low in untreated cells. 120×, 600×, 3000×, and SVNM treatment did not elevate IL-8 expression. We did not find significant differences between the DMSO controls at the three concentrations examined. We did not find significant differences between the ethanol controls. All the DTH-4 h exposures are significantly elevated compared to the DTH-1 h exposure groups. In the 0.1 µg/mL DTH group, 3000× and SNVM decreased IL-8 expression significantly compared to the DTH-4 h exposure. The same tendencies were present in the 0.25 µg/mL DTH group, where 3000× and SVNM significantly decreased IL-8 expression compared to DTH-4 h exposure. If we compare SVNM and dilution treatments, there is no significant difference at any concentration of DTH in IL-8 mRNA gene expression. 

**Figure 8:**
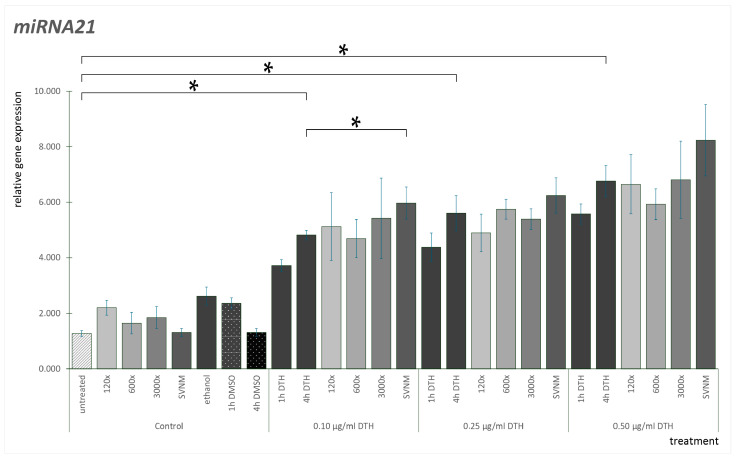
Relative gene expression level of miR-21 in HaCaT cells treated according to the experimental protocol. (* = *p* < 0.05). DTH—dithranol; 120×, 600×, 1200×-dilution of the tested organic matter isolate (Composition shown in Table 1); SVNM—Cell culture medium with 90% Szigetvár medicinal water content.

The miR-21 expression results are shown in Figure 8. If we analyze the gene expression pattern of miR-21, neither the vehicle controls nor the SVNM and isolates are elevated compared to the untreated control. We did not find significant differences between the DMSO controls at the three concentrations examined. We did not find significant differences between the ethanol controls. One h DTH treatment significantly elevated miR-21 expression at every concentration. Checking the DTH-4 h exposure, all were significantly higher than the corresponding DTH-1 h exposures. If we examine the SVNM and isolate exposures compared to the DTH-4 h exposures, at 0.5 µg/mL DTH, the 600× dilution reduced miR-21 expression, but the reduction is not statistically significant. At every DTH concentration, the SVNM exposure seemed to increase miR-21 expression compared to the DTH-4 h exposures. However, this is only significant at 0.1 µg/mL DTH.

## 4. Discussion

The overproduction of ROS and the malfunction of the physiological antioxidant responses play a crucial role in the pathogenesis of psoriasis [43]. MDA is derived from oxidative stress, which occurs when polyunsaturated fatty acids undergo peroxidation processes. MDA is widely used as a biomarker to measure oxidative stress from different biological samples, plasma or tissue samples of patients, or cell culture experiments [44]. Antioxidants can exert a protective effect in the epidermis against ROS, thus preventing the development of diseases [43]. DTH elevates MDA levels in a dose- and time-dependent manner. A 12.5 μM anthralin dose caused more than a twofold increase after 1 h and a 3.5-fold increase after 3 h of exposure [45]. Our findings correspond to this tendency: DTH elevated MDA levels significantly, and an increase can be seen between the DTH-1 h exposures and the DTH-4 h exposures. The increase is dose-dependent. The possible direct antioxidant effect of medicinal waters, or the modification of cells’ response to oxidative stress, has been discussed in various studies. Braga et al. demonstrated the antioxidant effects of sulfurous waters in cell-free systems and polymorphonuclear neutrophils (PMNs) [46]. Hakeem treated experimental rats with Mosul’s hot springs in Iraq and described antioxidant effects by measuring the MDA levels in various organs [32]. Joly et al., examining the thermal water through multiple parameters, found that exposing HaCaT cells to UVB radiation increased cell survival and improved oxidative parameters, including a reduction in lipid peroxidation levels, due to the thermal water [47]. Prandelli et al. investigated thermal water from Sirmione in primary human monocytes and samples from patients subjected to balneotherapy. Their results show that the combination of anti-inflammatory and antioxidant properties highlights that the thermal water exposure affected the regulation of antioxidant enzymes and increased the level of the anti-inflammatory cytokine IL-10 [48]. Szenczi et al. studied psoriasis patients treated with DTH who received spa therapy as a supplementary treatment. The patients’ plasma MDA levels decreased due to the spa treatment [10]. For our experiment, we chose a medicinal water with low sulfur and organic matter content. Szigetvár thermal water is a sodium-chloride and alkali-hydrogen-carbonate type of water [29]. Based on the MDA test, our results correlate with the literature data. The MDA level of untreated cells was elevated by DTH exposure, with a more significant increase observed after the first hour compared to the subsequent three hours. This suggests that oxidative damage processes occur early in the treatment. It must be highlighted that neither SVNM nor any dilution of the isolate elevates the MDA level significantly. According to our results, SVNM shows a protective effect against DTH-induced MDA elevation at all concentrations of DTH exposure. Interestingly, the organic isolates also showed significantly lower MDA levels in some experimental groups, suggesting that the organic matter content of the thermal water has the same effect as the water. However, the effect is smaller than that of thermal water. The full protective effect may be a result of the organic and inorganic matter combined.

### Effect of SVNM and Organic Isolates on the mRNA Expression of Selected Cytokines (IL-6, IL-8, TNF-α, GM-CSF)

It is well established that normal keratinocytes react to different types of oxidative stress (radiation or oxidizing chemicals, such as H_2_O_2_ or DTH). The noxa, through several signal pathways—via mitogen-activated protein (MAP) kinase signaling pathway or protein kinase c activation—leads to the induction, production, and release of pro-inflammatory mediators, including cytokines such as TNF-α, IL-6, IL-8, and GM-CSF [49,50,51]. The patient’s serum and psoriatic skin both showed elevated levels of many inflammatory cytokines. Serum levels of IL-17, IL-2, IL-18, and IFN-γ were substantially correlated with psoriasis [52]. Lange et al. investigated the effect of DTH on keratinocytes and measured the early inflammation response by expressing selected pro-inflammatory cytokines. In untreated cells, neither GM-CSF nor TNF-α mRNA transcripts were observed. IL-8 and IL-6 transcripts were expressed minimally. However, TGF-α, IL-1a, and IL-1b showed considerable constitutive expression. DTH exposure led to considerable increases in TNF-α, GM-CSF, IL-8, and IL-6 expressions. Following DTH treatment, GM-CSF, TNF-α, IL-6, and IL-8 significantly increased [35]. The overexpression of IL-6, IL-8, GM-CSF, and TNF-α was noticeable after 1 h and elevated considerably after 2–4 h. Similar results were reported by Wojciak et al. using an anti-inflammatory assay: H_2_O_2_ exposure elevated the expression of IL-6, IL-8, and TNF-α [36]. Zöller et al. treated HaCaT cells exposed to UV-B radiation with thermal spa waters (La Roche-Posay, Avène). Two thermal waters suppressed cell proliferation and damage, decreased ROS formation, and induced IL-6. They concluded that the investigated thermal spa waters can reduce inflammation-associated parameters. They also concluded that selenium and zinc are critical elements of the effect [53]. Mourelle et al. induced inflammation with lipopolysaccharides in HaCaT cells and found that La Solia thermal water exerted anti-inflammatory effects and that the induced levels of pro-inflammatory cytokines (IL-6, IL-1, TNF-α, NF-κB, and CCL1) were significantly attenuated by the water treatment [54]. 

T cells, particularly Th17 cells and the IL-23/IL-17 axis associated with them, play a key role in the pathogenesis of psoriasis, especially from a therapeutic perspective. The IL-23/IL-17 axis cytokines are involved in the initiation and maintenance of inflammation [55,56]. IL-23, produced by several cell types, including keratinocytes, stimulates Th17 cells, which release IL-17A. This cytokine has positive feedbacks on keratinocytes, leading to hyperproliferation and disturbed differentiation. Overall, activation of the IL-23 pathway promotes inflammatory cell recruitment to inflamed tissue. Clinical practice uses medications that block the cytokines of the IL-23/IL-17 axis with outstanding effectiveness [55,57,58,59]. In an imiquimod-induced mouse model, Lee et al. investigated the effect of Hae-Un-Dae hot spring mineral water on psoriasis. They observed inhibition of IL-23 and IL-17A in the lesions following balneotherapy. Because these cytokines are critical in the pathogenesis of psoriasis, their research suggests that balneotherapy could be a potential treatment for the disease [60]. Lee et al. also tested Yong-gung oncheon spring water on HaCaT cells to assess the balneotherapy’s anti-inflammatory and immunomodulatory effects. Their observations showed that the spring water reduced levels of IL-6, IL-8, TNF-α, IL-1α, and GM-CSF. Additionally, balneotherapy modulated the differentiation of CD4+ T cells into Th1, Th2, and Th17 cells [37]. Eliasse et al. investigated the effect of Avène thermal spring water (ATW) on human monocites. They found that ATW influenced the development of monocites into dendritic cells. The levels of IL-22 and IL-23 reduced. ATW also had an effect on Th17 responses by affecting their development, resulting in reduced IL-17 production [61]. Park et al. studied two types of hot springs, the NaCl-type and NaHCO_3_-type, common in South Korea. The HaCaT cells were stimulated with toll-like receptor 3 by polyinosinic-polycytidylic acid. The expression of inflammatory cytokines (IL-6, GM-CSF) increased. Cell proliferation and viability increased after spa treatment. The expression of inflammatory citokines did not change significantly. They stated that cytokines not only have pro-inflammatory effects but also beneficial effects on healing processes. In conclusion, balneotherapy has a beneficial effect, but the composition of the water must also be considered [62]. The reduction in inflammatory cytokines correlates with the anti-inflammatory effects. Our results are consistent with these findings, in which balneotherapy reduced the levels of molecular markers associated with inflammation. All of these results support the observed effects of medicinal waters and balneotherapy in practice. In our experiments, we demonstrated that Szigetvár medicinal water bears similar properties. As already seen with MDA concentrations, SVNM and isolate treatment showed significantly lower results than the 4 h control in several experimental settings. This was observed for TNF-alpha, IL-6, and GM-CSF in almost all experimental settings. In the case of IL-8, this was observed for the 3000× dilution and SVNM, and no difference was found for the 120× and 600× dilutions. No clear concentration-dependent effect can be stated for the dilutions. It was also demonstrated that if we isolate the organic matter content and test it separately, the organic matter content alone positively affected the upregulation of our tested cytokines as well. SVNM consistently showed better results than the organic isolates, suggesting that the organic and inorganic content together is responsible for the full protective effect. The 3000× dilution, which has the same organic matter concentration as SVNM, showed similar but lesser results in decreasing elevated cytokine levels than the SVNM. 600× dilution in most of the cases decreased cytokine levels significantly. On the other hand, 120× dilution failed to protect against DTH-induced cytokine increase in many cases. We must highlight that 600× and 120× dilutions have no practical importance. This isolate is recommended to be used at 2–3000× dilution for balneotherapy treatment; 3000× dilution is equal to the organic matter content of the native medicinal water. Testing of 120× and 600× dilutions showed neither elevated MDA nor significantly elevated cytokine levels compared to the DTH-1 h and DTH-4 h exposure groups. This is a partial proof of the safety of the isolate. MicroRNAs (miRNAs) are small, non-coding RNAs that influence numerous physiological and pathological processes. They are post-transcriptional regulators of gene expression that are bound to and interact with mature mRNAs in a sequence-specific manner [63]. miR-21 is one of the critical regulators of ROS homeostasis and responses to non-physiological oxidative stress. Common oxidative noxae, such as H_2_O_2_ or UV radiation, upregulate the expression of miR-21 and mediate the upregulation of ROS-induced signaling pathways [39,64]. Abdallah et al. investigated the effect of miR-21-3p in psoriasis pathogenesis. miR-21 is one of the critical miRNAs in psoriatic processes, both as a marker due to its upregulation and as a therapeutic target. They also found that IL-22 induces miR-21-3p expression, interfering with other inflammatory cytokines; IL-1β is upregulated, and IL-6 and IL-4 are downregulated [38]. miR-21 inhibits transforming growth factorβ (TGF-β) signaling. Overexpression of TGF-β can be measured in psoriatic lesions and serum samples. TGF-β overexpression correlates with skin inflammation, keratinocyte proliferation, inhibition of apoptosis, and T-cell activation [40]. miR-21 is connected with TNF-α signaling pathways through negative feedback. TNF-α activates STAT3, a potent transcriptional activator of miR-21. miR-21 has a crucial role in skin damage and wound healing processes involving normal keratinocytes [65]. miR-21 negatively regulates tumor suppressor programmed cell death 4 (PDCD4). PDCD4 inhibits cell proliferation and can increase NF-κB activity, resulting in TNF-α increase and inflammation. It was shown that miR-21 inhibition increased PDCD4 expression. This mechanism explains that elevated miR-21 levels can contribute to defensive mechanisms against oxidative stress, cell repair, and survival [66,67,68]. Our results are in harmony with the literature. MiR-21 expression is increased by DTH exposure compared to untreated cells. SVNM and isolates themselves do not increase miR-21 expression compared to the untreated cells. SVNM seemingly elevated miR-21 levels compared to the 4 h positive DTH controls, but this was only significant in the case of 0.1 µg/mL DTH exposure. Increases in miR-21 levels correlate with elevated TNF-α levels and the activation of cellular defense mechanisms. SVNM and isolate treatments reduced TNF-α mRNA expression, but this was not directly reflected in changes in mir-21 expression.

## 5. Conclusions

Our results show that Szigetvár medicinal water can reduce oxidative stress caused by dithranol in HaCaT cells. Balneotherapy with Szigetvár medicinal water might be useful to counteract the side effects of DTH therapy on intact skin. Our investigation confirmed for the first time that the organic isolate of Szigetvár medicinal water also exhibits the same protective function, supporting the organic theory of medicinal waters.

## Figures and Tables

**Table 1:** Composition of the dilution series of the organic isolate.

Dilution	DMEM Added to 100 μL of Isolate (mL)
6×	0.5
12×	1.1
30×	2.4
40×	3.9
60×	5.9
120×	11.9
250×	24.9
300×	29.9
600×	59.9
1500×	149.9
3000×	299.9

**Table 2:** Experimental design.

Treatment		Controls (3 h)
#1		120×
#2		600×
#3		3000×
#4		SVNM
#5		vehicle controls (ethanol–3 concentrations)
#6		vehicle controls (DMSO–3 concentrations)
#7		negative control
	dithranol treatment (µg/mL^−1^ h)	post dithranol treatment (3 h)
#8	0.1	-
#9	0.1	DMEM
#10	0.1	120×
#11	0.1	600×
#12	0.1	3000×
#13	0.1	SVNM
#14	0.25	-
#15	0.25	DMEM
#16	0.25	120×
#17	0.25	600×
#18	0.25	3000×
#19	0.25	SVNM
#20	0.5	-
#21	0.5	DMEM
#22	0.5	120×
#23	0.5	6000×
#24	0.5	3000×
#25	0.5	SVNM

120×, 600×, 3000×–dilutions of the organic matter isolate, SVNM—Cell culture medium with 90% Szigetvár medicinal water content.

## Data Availability

Data will be made available upon request due to the fact that this manuscript is part of a multiple study.
